# Functional and radiographic assessments of post-traumatic asymmetrical ankle osteoarthritis treatment using supramalleolar osteotomies

**DOI:** 10.1007/s00590-023-03773-x

**Published:** 2023-11-09

**Authors:** Wael El-Adly, Faisal Fahmy Adam, Mohamed Sayed Kamel, Ahmed E. Osman

**Affiliations:** 1 Orthopedic Surgery Department, Assiut University, Assiut, Egypt; 2 Orthopedic Surgery Department, Assiut University Hospital, University Street, Assiut, 71515 Egypt

**Keywords:** Asymmetrical ankle osteoarthritis, Supramalleolar osteotomies, Fibular osteotomy, AOFAS ankle–hindfoot score

## Abstract

**Purpose:**

This study's objective is to assess the effectiveness of supramalleolar osteotomies (SMOT) in the treatment of asymmetrical ankle osteoarthritis in terms of the improvement in alignment assessed radiographically and its impact on symptoms measured by the American Orthopedic Foot and Ankle Society ankle–hindfoot score (AOFAS ankle–hindfoot score).

**Methods:**

Twelve patients participated in this prospective observational case series study. Standing anteroposterior, true lateral, and mortise views radiographs were taken. For preoperative planning, the lateral distal tibial angle (LDTA), talar tilt (TT), talocrural angle (TCA), and anterior distal tibial angle (ADTA) were all measured. A medial opening wedge osteotomy mainly was used to treat the varus arthritis ankle. A further oblique fibular osteotomy is frequently necessary.

**Results:**

The male-to-female ratio was 3:1, and the mean age of the patients was 26.25 ± 13.032 years. There were highly statistically significant differences between pre-and post-operative AOFAS ankle–hindfoot score (*p* < 0.001). Statistically significant differences concerning ankle range of motion (*p* = 0.002, < 0.001) of dorsiflexion and planter flexion were detected. Comparison between pre-and post-operative patients' radiology characteristics shows statistically significant differences concerning TT (*p* = 0.016) and LDTA (*p* = 0.046).

**Conclusion:**

SMOT is very effective in the treatment of post-traumatic ankle osteoarthritis and postpones ankle fusion or total ankle replacements. This surgery significantly improves functional and radiological outcomes as well as the range of motion in the ankle. Meticulous preoperative planning by radiological measurements of different angles around the ankle is considered the crucial factor in success of that operation.

## Introduction

According to reports, post-traumatic ankle arthritis accounts for 70–80% of all cases of ankle arthritis, making it the most prevalent cause [1]. Initial cartilage damage, malreduction, nonunion, infections, and instability are factors that cause post-traumatic ankle arthritis [2, 3]. The degenerative changes frequently manifest asymmetrically in patients with post-traumatic ankle osteoarthritis (OA), along with a concurrent varus or valgus deformity of the hindfoot [4, 5]. Non-operative treatments, like, anti-inflammatory drugs, activity modification, orthotics, wedges, and shoe modifications are almost typically ineffective [6]. The typical treatment for ankle arthritis is ankle arthrodesis, but it has the drawback of causing adjacent joint arthritis in the hindfoot and forefoot due to significant loss of motion in the ankle [7]. In aged, low-demand patients with neutral alignment and intact stabilizing ligaments total ankle replacements (TAR) are indicated [8]. In recent years, supramalleolar osteotomies (SMOT) have emerged as a useful alternative for the treatment of ankle osteoarthritis [9]. Sagittal and rotational deformities, as well as varus and valgus, have been treated with SMOT [10]. The objective of the osteotomy is to prevent the occurrence or stop the progression of ankle arthritis by correction of the mechanical axis, reducing pain, and redistributing the joint forces by shifting weight-bearing to intact cartilage [11]. The SMOT has been successful in preventing the need for ankle arthrodesis or TAR by protecting the joint. Young active patients with deformity or mild-to-moderate arthritis should have the option of trying realignment with an SMOT before looking into fusion arthroplasty options, it is believed, because complications are uncommon and bone loss is negligible [11]. This study's objective is to assess the effectiveness of SMOT in the treatment of asymmetrical ankle OA in terms of the improvement in alignment assessed radiographically and its impact on symptoms measured by the American Orthopedic Foot and Ankle Society ankle–hindfoot score (AOFAS ankle–hindfoot score).

## Patients and methods

This prospective observational case series study was conducted in the orthopedic surgery department in a University Hospital between 2019 and 2021. Patients between the ages of 15 and 60 who had varus or valgus ankle deformity with an asymmetric osteoarthritis (OA) involving less than 50% of the articular surface and clinical symptoms like walking pain, limitations on daily and recreational activities were included in this study. The following conditions were excluded from the study: severe hindfoot instability that is uncontrollable, severe vascular or neurologic deficit in the affected extremity, chronic liver disease, renal failure, rheumatic disease, morbid obesity, and with condition altered bone quality due to medication, large cysts, osteoporosis, and osteopenia. The study was carried out in accordance with the Declaration of Helsinki's ethical principles and has been approved by the institutional review board of the author's affiliated institution. The patients gave written informed consent to take part in the study.

**Preoperative assessment**: Medical history was taken, and clinical examinations were done including a clinical assessment of the ankle, hindfoot, and forefoot in a standing position to assess the type of the deformity and swelling of the ankle joint. Palpation to detect the site of tenderness, assess the integrity of the medial and lateral collateral ligaments, and the passive and active range of motion (ROM) of ankle and subtalar joints. Radiographs of the true lateral, mortise, and standing anteroposterior (AP) were taken. Radiographic signs of post-traumatic asymmetrical ankle OA, such as subchondral sclerosis, osteophytes, asymmetric joint space narrowing, and cartilage degeneration can be seen, and a varus or valgus tilt in the subtalar and/or ankle joint can also be evaluated. Saltzman view was used to evaluate the hindfoot's overall alignment. A CT scan was used to identify occult arthritis in the ankle or other joints; MRI was done in some selected cases to rule out stress fractures, soft tissue infections, and tumors. It was helpful also in detection of the extent of ligamentous injury.

**Preoperative planning:** lateral distal tibial angle (LDTA), talar tilt (TT), talocrural angle (TCA), and anterior distal tibial angle (ADTA) were all measured. In case of abnormal LDTA and ADTA values, the center of rotation and angulation (CORA) of the deformity of the distal tibia using the anatomical axis planning was determined.

### Surgical technique

**Fig. 1:**
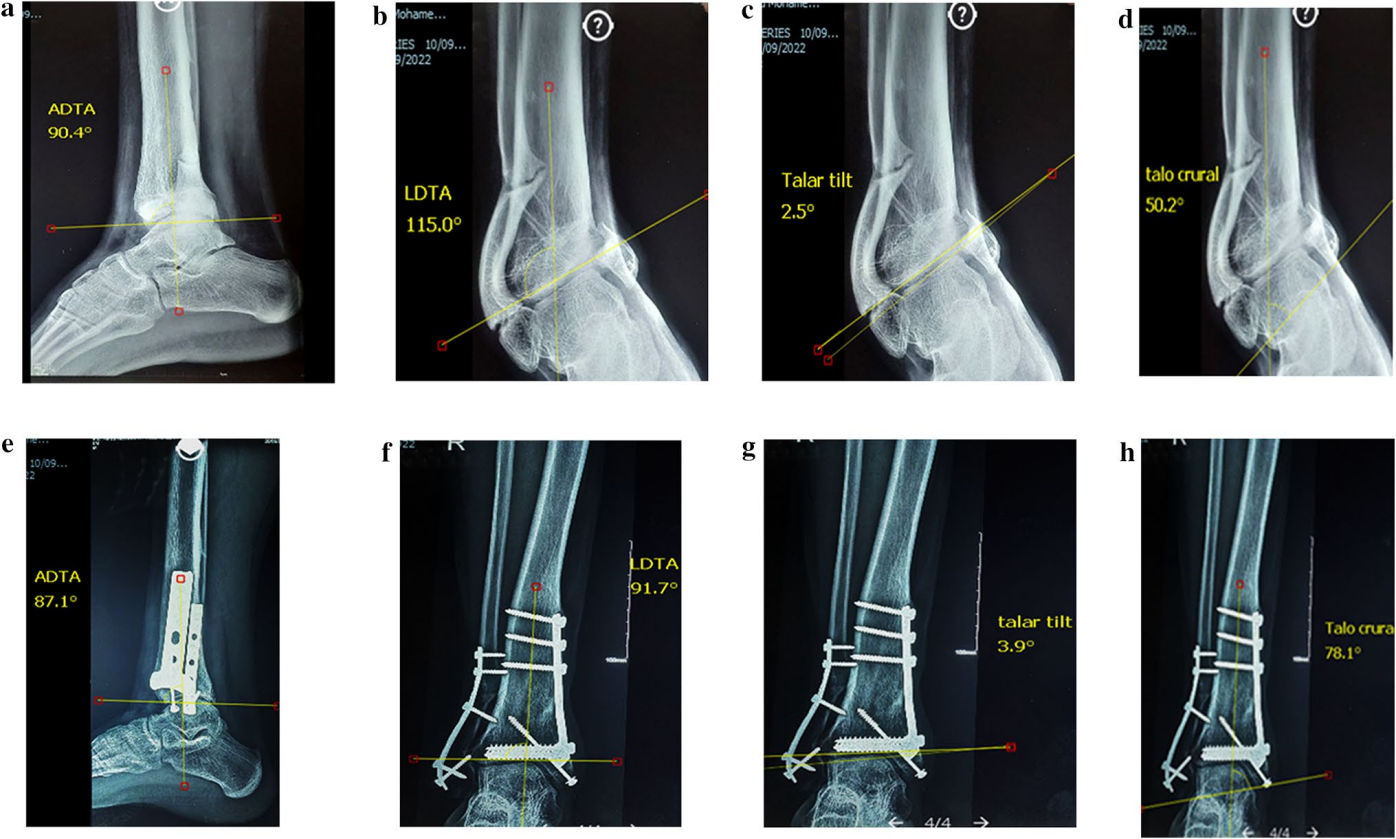
A Male patient 22 years old presented after one year from trauma by post-traumatic right ankle OA with varus deformity and nonunion of distal third fibular fracture and medial malleolus. Medial wedge opening supramalleolar osteotomy with bone graft, fibular plating, and screw fixation of the medial malleolus were done. **a**–**d** Preoperative X-rays show degrees of anterior distal tibial angle (ADTA), lateral distal tibial angle (LDTA), talar tilt (TT), and talocrural angle (TCA). **e**–**h** X-rays show degrees of ADTA, LDTA, TT, and TCA after 8 weeks with the union of supramalleolar osteotomy, fibular fracture and medial malleolus.

**Fig. 2:**
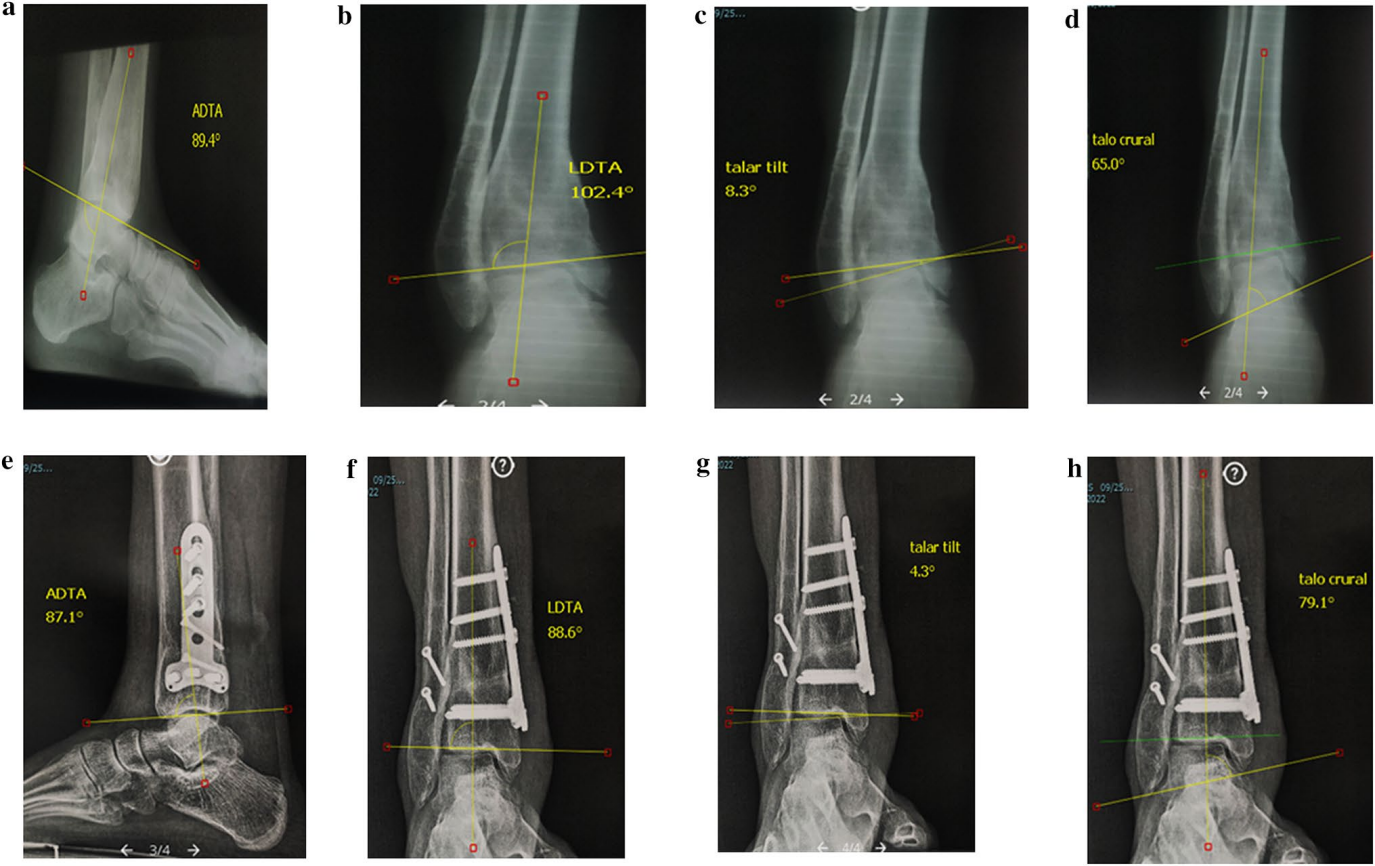
A Male patient 28 years old presented after 2 years from trauma by post-traumatic right ankle OA with varus deformity. Medial wedge opening supramalleolar osteotomy with bone graft, and fibular osteotomy were done. **a**–**d** Preoperative X-rays show degrees of anterior distal tibial angle (ADTA), lateral distal tibial angle (LDTA), talar tilt (TT), and talocrural angle (TCA). **e**–**h** X-rays show degrees of ADTA, LDTA, TT, and TCA after 9 weeks with union of supramalleolar, and fibular osteotomies
- **Valgus Deformity**: A medial-based wedge closing osteotomy was done through medial approach to the distal tibia. Overcorrection of the LDTA by 2–4° in varus direction was aimed by such osteotomy. In accordance with the preoperative plan, a low cut was made for resection of the tibial bone, which was parallel to and within 15 mm proximal to the joint space giving adequate distance for the distal fixation of the used plate. The upper cut was done obliquely in such way to remove the preplanned length of the base of the wedge from the medial tibial cortex. Placement of two guide wires in the site and the direction of the planned osteotomy was done under image intensifier guidance to ensure accurate bone resection. The osteotomy was then made with a wide saw blade and forced to be shut manually. To achieve a stable fixation, a rigid locked plate fixation is used.
- **Additional Procedures**: (1) Fibular Osteotomy (FO): Oblique or Z-form fibular osteotomy (FO) was frequently necessary. The FO is carried out using a separate lateral approach. It was indicated if the talus was not properly repositioned to the medial malleolus after the SMOT had been performed in patients with a congruent or incongruent deformity. This osteotomy was stabilized with one plate or two screws according to lateral soft tissue condition (Fig. 2). (2) Medial or lateral sliding calcaneus osteotomy aiming for realigning the hindfoot with painless mobile subtalar joint. (3) Subtalar joint fusion was used for correction of hindfoot residual varus or valgus deformity associated with painful degenerative changes of the subtalar joint. (4) Proximal dorsal wedge closing osteotomy of first metatarsal to correct the forefoot pronation. (5) Reconstruction of the lateral collateral ligament (LCL) of the ankle was done by Broström-Gauld operation. Arthroscopic assessment of the ankle joint was done in one case for assessment the extent of cartilage damage and severity of the LCL injury. This was necessary to stabilize the talus in a corrected position within the ankle mortise after bony correction. (6) Tendons transfer around the ankle was needed to restore the muscular balance. Total split tibialis posterior tendon transfer to the peroneus brevis and the tibialis anterior tendons in the dorsum of the foot was used in the cases of weakness of ankle pronation and dorsiflexion.
- **Post-operative Protocol:** Patient follow-up at the time of suture removal after 2 weeks. Radiographic examinations were done after 1.5 months, 3 months, and 1 year for assessment of union and angles mentioned before. The patients were immobilized in a plaster cast for 6 weeks with adequate thromboembolic prophylaxis. Protected full weight-bearing in walking boots for further 6 weeks is advised. Full weight-bearing was allowed after healing of osteotomies. Rehabilitation exercises like passive and active mobilization, coordination and proprioception of muscular activity, muscle strengthening, and gait training were started after the 6th week post-operative. The rehabilitation program was tailored for each patient according to the bony and soft tissue procedure done. Assessment of AOFAS ankle–hindfoot score for pain, function, and alignment was done in the final visit.

- **Varus Deformity**: SMOT was done through a medial approach to the distal tibia. All patients received treatment with a medial opening wedge osteotomy. When performing a medially-based open wedge osteotomy, if the CORA level was at the level of the tibial joint surface or within 1.5 cm proximal to it, the designed osteotomy made 15 mm above the joint space to allow for minimal osteosynthesis, improve union, and avoid bayonet deformities. The goal of the osteotomy is to obtain overcorrection about 4 degrees of valgus of the LDTA to compensate for the cartilage degeneration in the medial tibiotalar joint. The preexisting varus deformation forces that affect the soft tissue are also offset by this overcorrection (Fig. 1). If site of the CORA was higher than the 2.5 cm proximal to tibial plafond, the designed osteotomy was done through it running obliquely from distal medial to proximal lateral applying osteotomy rule one for deformity correction (Fig. 2).

### Statistical analysis

Data collected coded, entered, and analyzed using Microsoft Excel software. The Statistical Package for the Social Sciences (SPSS version 21.0) program was then used to import the data and perform analysis according to the type of data. The quantitative continues group is represented by mean ± SD, and the qualitative variables represent the number and percentage. The following tests were used to determine the significance of differences; difference and association of qualitative variables by Chi-square test (X^2^), and differences between quantitative independent groups by *t*-test. The *p* value was set at < 0.05 for significant results and < 0.001 for high-significant results.

## Results

The demographic and operation data of the studied patients are presented in Table 1. Table 2 compares the pre- and post-operative patient's AOFAS ankle–hindfoot scores. It reveals highly statistically significant differences in all score items, including pain, function, and alignment (*p* = 0.001, < 0.001, 0.008) with total range (*p* < 0.001). Comparing the range of motion (ROM) of the ankle in pre- and post-operative patients, Table 3 reveals highly statistically significant variations in dorsiflexion and planter flexion (*p* = 0.002, 0.001). Table 4 shows a comparison between pre-and post-operative patients' radiology characteristics and it shows statistically significant differences concerning TT (*p* = 0.016) and LDTA (*p* = 0.046). Alignment in preoperative time shows that 11 patients (91.7%) had varus alignment one of them had previous subtalar fusion, and only one patient (8.3%) had valgus alignment. Post-operatively, all patients had neutral alignment (100%). The correction of the hindfoot alignment was statistically significant (*p* = 0.008).Seven patients (58.3%) had a union after 6–8 weeks, while five patients (41.6%) had a union after 8–10 weeks. No serious complications were detected in our cases.

**Table 1:** Demographic and operation data of patients

Characteristics mean ± SD or *n* (%)	Patients (*n* = 12)
Age (years)	26.25 ± 13.032
Sex	
Male	9 (75)
Female	3 (25)
BMI	22.33 ± 3.495
Side	
Right	6 (50)
Left	6 (50)
Diagnosis	
Post-traumatic ankle OA with varus deformity	9 (75)
Post-traumatic ankle OA with subtalar fusion and varus deformity	1 (8.3)
Old neglected fracture distal tibia with varus deformity, and OA ankle	1 (8.3)
Post-traumatic ankle with valgus deformity	1 (8.3)
Operation	
Supramolecular osteotomy	12 (100)
Fibular osteotomy	9 (75)
Calcaneal osteotomy	1 (8.3)
Medial arch osteotomy	1 (8.3)
Tendon transfer	1 (8.3)
Arthroscopic LCL repair	2 (16.6)
Follow-up duration (months)	17.25 ± 3.671

*BMI* body mass index, *OA* osteoarthritis, *LCL* lateral collateral ligament

**Table 2:** Comparison between pre- and post-operative patients' ROM

Characteristics mean ± SD	Preoperative	Post-operative	*p* value
Dorsiflexion	1.67 ± 2.462	7.92 ± 2.575	0.002
Planter flexion	21.67 ± 9.129	32.50 ± 8.118	< 0.001

*ROM* range of motion

**Table 3:** Comparison between pre-and post-operative patient's radiology characteristics

Characteristics mean ± SD	Preoperative	Post-operative	*p* value
ADTA	84.40 ± 8.505	84.56 ± 4.324	0.948
LDTA	100.42 ± 14.535	90.00 ± 3.403	0.046
TT	4.98 ± 1.938	3.45 ± 0.626	0.016
TCA	50.2–95.2	72.9–91.5	0.079

*ADTA* anterior distal tibial angle, *LDTA* lateral distal tibial angle, *TT* talar tilt, *TCA* talocrural angle

**Table 4:** Comparison between pre- and post-operative AOFAS ankle–hindfoot score

Characteristics mean ± SD	Preoperative	Post-operative	*p* value
Pain	19.17 ± 9.962	31.67 ± 5.774	0.001
Function	22.83 ± 7.259	41.08 ± 4.757	< 0.001
Alignment	2.50 ± 3.371	6.67 ± 2.462	0.008
Total	44.50 ± 14.532	79.42 ± 9.904	< 0.001

*AOFAS* American Orthopedic Foot and Ankle Society

## Discussion

Ankle OA is a degenerative disease in which the distal tibial articular surface frequently exhibits angular deformity [12]. According to Salter et al., there is a correlation between uneven pressure on the lower extremities' articular surface and cartilage degeneration, which may cause and hasten the development of OA [13]. The objectives of SMOT are to realign the hindfoot, and move the ankle joint under the weight-bearing axis, and normalize the direction of the triceps surae force vector [5]. In the current study we evaluated the functional and radiological results of SMOT in treatment of post-traumatic asymmetrical ankle arthritis. Previous research demonstrated that SMOT can enhance patients' functional outcomes by reducing pain, enhancing function, and correcting varus deformity with positive outcomes [14–18].These results were withstand with ours as the current study detected highly statistically significant differences between pre-and post-operative AOFAS hindfoot score items. As regards to our patient's ROM of the ankle, there was a highly statistically significant increase between pre- and post-operative time regarding dorsiflexion and plantarflexion with no significant differences of subtalar ROM. In agreement of our result Liang et al. [18], revealed that with the SMOT, the ROM of the ankle joint was significantly increased, but in contrast, Zhao et al. [19] and Hongmou et al. [20] revealed that the ROM of the ankle was non-significantly increased in supramalleolar osteotomy. The distal portion of the tibia was rotated with the lateral cortex of the tibia serving as the rotational center while tibial osteotomy was gradually performed. Since the syndesmosis is still intact in this situation, it may be necessary to perform a fibular osteotomy (FO) in order to allow for the medial opening of the tibia. They served the same purposes in relieving lateral stress when the osteotomy site was opened and enabling the distal tibial to rotate centered on the lateral cortex. Due to the anatomy of the talus dome, which has a wide anterior and narrow posterior, SMOT without FO may result in a narrowed ankle mortise, which could limit the ankle joint's dorsiflexion [18]. Also correcting the angle of the ankle joint is made easier by FO [9, 14]. The degree of the deformity is taken into consideration when deciding whether to perform a concomitant fibular osteotomy, which is typically decided intraoperatively. In our experience, deformity of more than 10 degrees generally necessitates fibular correction. If the reduction of the talus is prevented by the fibula, a separate lateral incision must be made in order to add a FO because the medial displacement of the talus was better reduced in the SMOT with FO. In our study FO were done in 75% of cases with statistically significant differences concerning pre-and post-operative patient's radiology. As regards to patient's radiology characteristics, our result revealed a highly statistically significant decrease between pre- and post-operative TT. A statistically significant decrease in LDTA between pre- and post-operative time is also seen. However, recent researches suggest that asymmetric arthritis of the ankle joint in the most cases is not only due to a single plane deformity but may include a complex instability pattern involving the ankle joint, the neighboring joints, and the surrounding stabilizing soft tissues. Therefore, these patients may require a correction of the angle of the distal tibial articular joint surface (TAS) and also need further procedures for the adjacent joints, ligaments, and tendons [21–23]. The TT that distinguishes varus ankle osteoarthritis is brought on by the tibiotalar joint's incongruence rather than a bony deformity. A LCL of the ankle joint could be the initial cause of the increased TT angle. However, this will cause the center of the talus and joint loading axis to shift medially [12], which will increase the tension and swelling of the lateral soft tissue, thereby worsening the deformity. According to some authors, SMOT could significantly reduce the TT angle [21, 24]. Furthermore, Liang et al. [18] revealed that all of the radiological parameters were significantly improved in supra malleolar osteotomy with and without FO groups. Except when there was a post-traumatic leg length discrepancy of more than 1 cm and poor soft tissue conditions like existing scars or skin injury, the lateral closing wedge osteotomy was the standard procedure. The lateral closing wedge makes it possible to easily access the fibula, avoid the need to insert grafts, increase the construct's inherent stability, and prevent medial soft tissue compromise. For the treatment of the varus deformity, tibial open wedge osteotomy has recently replaced lateral closing wedge osteotomy as the preferred method. In many reported lateral closing wedge procedures, the complexity of the anterolateral compartment of the leg emerges as the primary risk factor for lateral muscle weakness [25]. Nine out of 11 patients with varus deformities were corrected by open wedge osteotomy without complications and with statistically significant differences of AOFAS ankle–hindfoot score. There are a few limitations on this study. First, a small sample size, a brief period of follow-up, and numerous additional surgical procedures were required for the deformity's proper correction, with it being challenging to predict how these factors would affect the final results. To address both positive and negative predictors influencing long-term success following this surgery, more long-term studies are advised.

## Conclusion

SMOT is very effective in the treatment of post-traumatic ankle OA and postpones ankle fusion or TAR. It's recommended in early to mid-stage post-traumatic asymmetric varus and valgus ankle OA. To identify the patients who would benefit from this treatment, preoperative evaluation is required. Meticulous preoperative planning by radiological measurements of different angles around the ankle is considered the crucial factor in success of that operation. This surgery results in significant pain relief, functional and radiological improvements, and a significant increase in ankle ROM. All cases achieved bony unions with mild complications.

## References

[1] Saltzman CL, Salamon ML, Blanchard GM (2005). Epidemiology of ankle arthritis: report of a consecutive series of 639 patients from a tertiary orthopedic center. Iowa Orthop J.

[2] Stufkens SA, Knupp M, Horisberger M (2010). Cartilage lesions and the development of osteoarthritis after internal fixation of ankle fractures: a prospective study. J Bone Joint Surg Am.

[3] Harrington KD (1979). Degenerative arthritis of the ankle secondary to long-standing lateral Ligament instability. J Bone Joint Surg Am.

[4] Horisberger M, Valderrabano V, Hintermann B (2009). Posttraumatic ankle osteoarthritis after ankle-related fractures. J Orthop Trauma.

[5] Knupp M, Stufkens SA, Bolliger L (2011). Classification and treatment of supra malleolar deformities. Foot Ankle Int.

[6] Harstall R, Lehmann O, Krause F (2007). Supramalleolar lateral closing wedge osteotomy for the treatment of varus ankle arthrosis. Foot Ankle Int.

[7] Pagenstert G, Knupp M, Valderrabano V (2009). Realignment surgery for valgus ankle osteoarthritis. Oper Orthop Traumatol.

[8] Swords MP, Nemec S (2007). Osteotomy for salvage of the arthritic ankle. Foot Ankle Clin.

[9] Ahn TK, Yi Y, Cho JH (2015). A cohort study of patients undergoing distal tibial osteotomy without fibular osteotomy for medial ankle arthritis with mortise widening. J Bone Joint Surg Am.

[10] Hintermann B, Knupp M, Barg A (2013). Joint-preserving surgery of asymmetric ankle osteoarthritis with peritalar instability. Foot Ankle Clin.

[11] Knupp M (2017). The use of osteotomies in the treatment of asymmetric ankle joint arthritis. Foot Ankle Int.

[12] Zhao H, Liang X, Li Y (2016). The role of fibular for supra malleolar osteotomy in the treatment of varus ankle arthritis: a biomechanical and clinical study. J Orthop Surg Res.

[13] Salter RB, Field P (1960). The effects of continuous compression on living articular cartilage. J Bone Joint Surg Am.

[14] Cheng YM, Huang PJ, Hong SH (2001). Low tibial osteotomy for moderate ankle arthritis. Arch Orthop Trauma Surg.

[15] Knupp M, Barg A, Bolliger L (2012). Reconstructive surgery for overcorrected clubfoot in adults. J Bone Joint Surg Am.

[16] Krähenbühl N, Akkaya M, Deforth M (2019). Extraarticular supramalleolar osteotomy in asymmetric varus ankle osteoarthritis. Foot Ankle Int.

[17] Choi JY, Kim KW, Suh JS (2020). Low tibial vulgarization osteotomy for more severe varus ankle arthritis. Foot Ankle Int.

[18] Liang JQ, Wang JH, Zhang Y (2021). Fibular osteotomy is helpful for talar reduction in the treatment of varus ankle osteoarthritis with supra malleolar osteotomy. J Orthop Surg Res.

[19] Zhao HM, Wen XD, Zhang Y (2019). Supramalleolar osteotomy with medial distraction arthroplasty for ankle osteoarthritis with talar tilt. J Orthop Surg Res.

[20] Hongmou Z, Xiaojun L, Yi L (2016). Supramalleolar osteotomy with or without fibular osteotomy for varus ankle arthritis. Foot Ankle Int.

[21] Pagenstert GI, Hintermann B, Barg A (2007). Realignment surgery as an alternative treatment of varus and valgus ankle osteoarthritis. Clin Orthop Relat Res.

[22] Stamatis ED, Cooper PS, Myerson MS (2003). Supramalleolar osteotomy for the treatment of distal tibial angular deformities and arthritis of the ankle joint. Foot Ankle Int.

[23] Takakura Y, Tanaka Y, Kumai T et al (1995) Low tibial osteotomy for osteoarthritis of the ankle. Results of a new operation in 18 patients. J Bone Joint Surg Br 77(1): 50–54. 7822395

[24] Kobayashi H, Kageyama Y, Shido Y (2016). Treatment of varus ankle osteoarthritis and instability with a novel mortise-plasty osteotomy procedure. J Foot Ankle Surg.

[25] Chilmi MZ, Desnantyo AT, Widhiyanto L (2020). Low tibial and fibular osteotomy for treating varus-type post-traumatic ankle osteoarthritis: a case report. Malays Orthop J.

